# Supplementary material for: Sequences of purchases in credit card data reveal lifestyles in urban populations
Source: Nat Commun. 2018 Aug 20;9:3330. doi: 10.1038/s41467-018-05690-8 (PMC6102281; doi:10.1038/s41467-018-05690-8)
Supplement: Supplementary file 1 — Supplementary Information [file 41467_2018_5690_MOESM1_ESM.pdf]

Supplementary Information

Sequence of purchases in credit card data reveal life styles in urban populations

Riccardo Di Clemente[1,2], Miguel Luengo-Oroz[3], Matias Travizano[4], Sharon Xu[1], Bapu Vaitla[5], Marta C. González[1,6,7*]

[1]*Department of Civil and Environmental Engineering, Massachusetts Institute of Technology, Massachusetts Avenue 77, MA 02139 Cambridge, USA*
[2]*University College London, The Bartlett Centre for Advanced Spatial Analysis, London, WC1E 6BT, United Kingdom*
[3]*United Nations Global Pulse, 46th St & 1st Ave, New York, NY 10017, USA*
[4]*GranData, 550 15th St. Suite 36C San Francisco, CA 94103, USA*
[5]*Department of Environmental Health, Harvard University, 677 Huntington Avenue Boston, MA 02115, USA*
[6]*Department of City and Regional Planning, Berkeley, CA 94720-1820, USA*
[7]*Lawrence Berkeley National Laboratory, 1 Cyclotron Road, Berkeley CA 94720-1820, USA*

[*]Corresponding author. *E-mail address*: martag@mit.edu

Figures

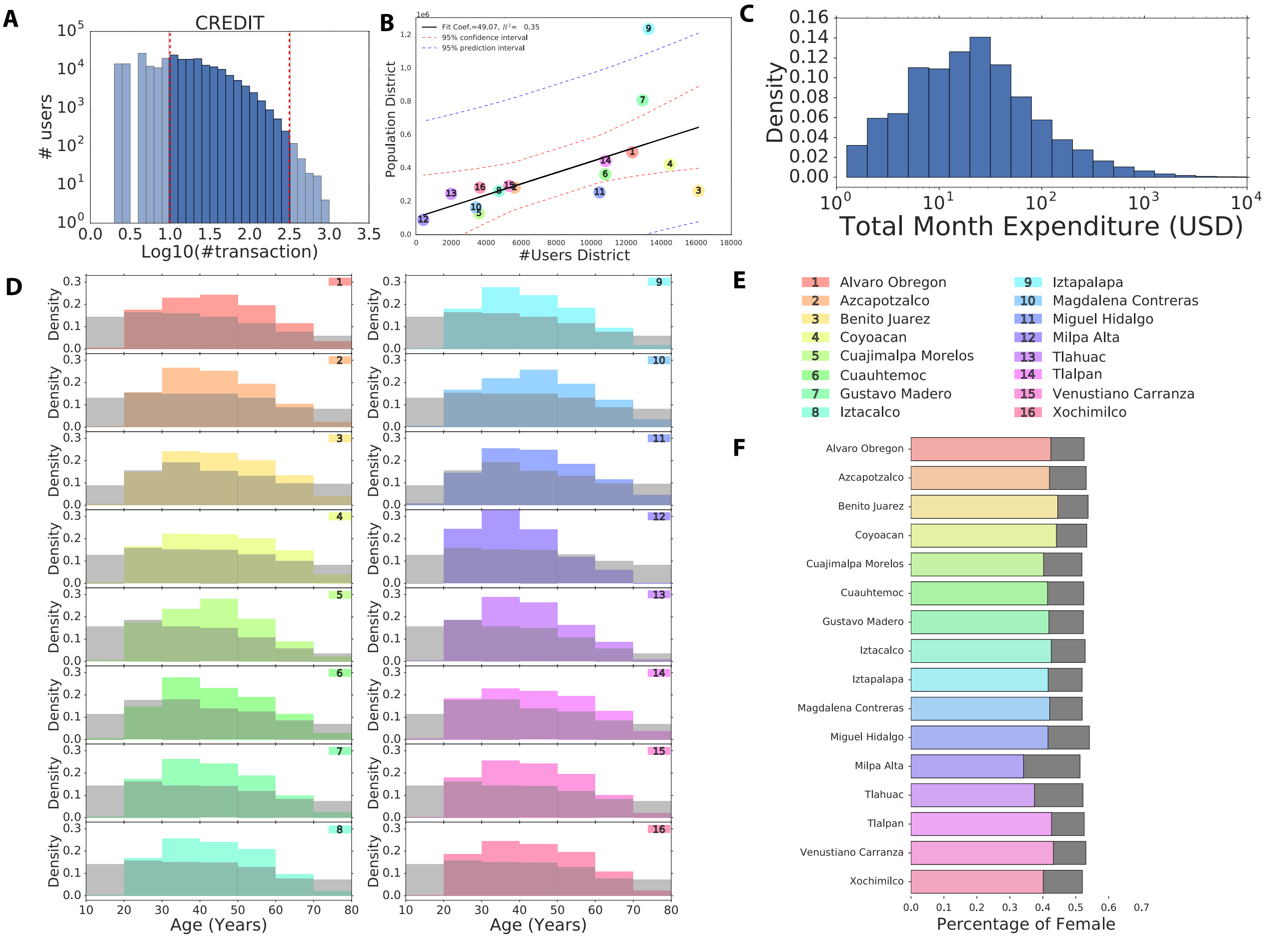

Supplementary Figure 1: **(A)** Histogram of the user transaction number in the CCRs, the 150,000 users selected for the analysis are those with more than 10 transactions and less than 300. **(B)** Relation between the district population (”Source: INEGI, Intercensal Survey 2015”) and the number of user in our datasets, using the same color map as Fig. 1 in the paper (each color represents a city district Fig. 1A main text, the districts legend in Fig. S1s). **(C)** Distribution of overall users’ monthly expenditure in USD. **(D)** Comparison between district users of CCR and district population from Census Data in gray (”Source: INEGI, Intercensal Survey 2015”). **(E)** Mexico City Color legend district for Fig. 1A of the main text and figures S1b-S1d-S1F. **(F)** Female percentage usage of credit card (by district) CCRs, in comparison with the female percentage from census in gray (”Source: INEGI, Intercensal Survey 2015”).

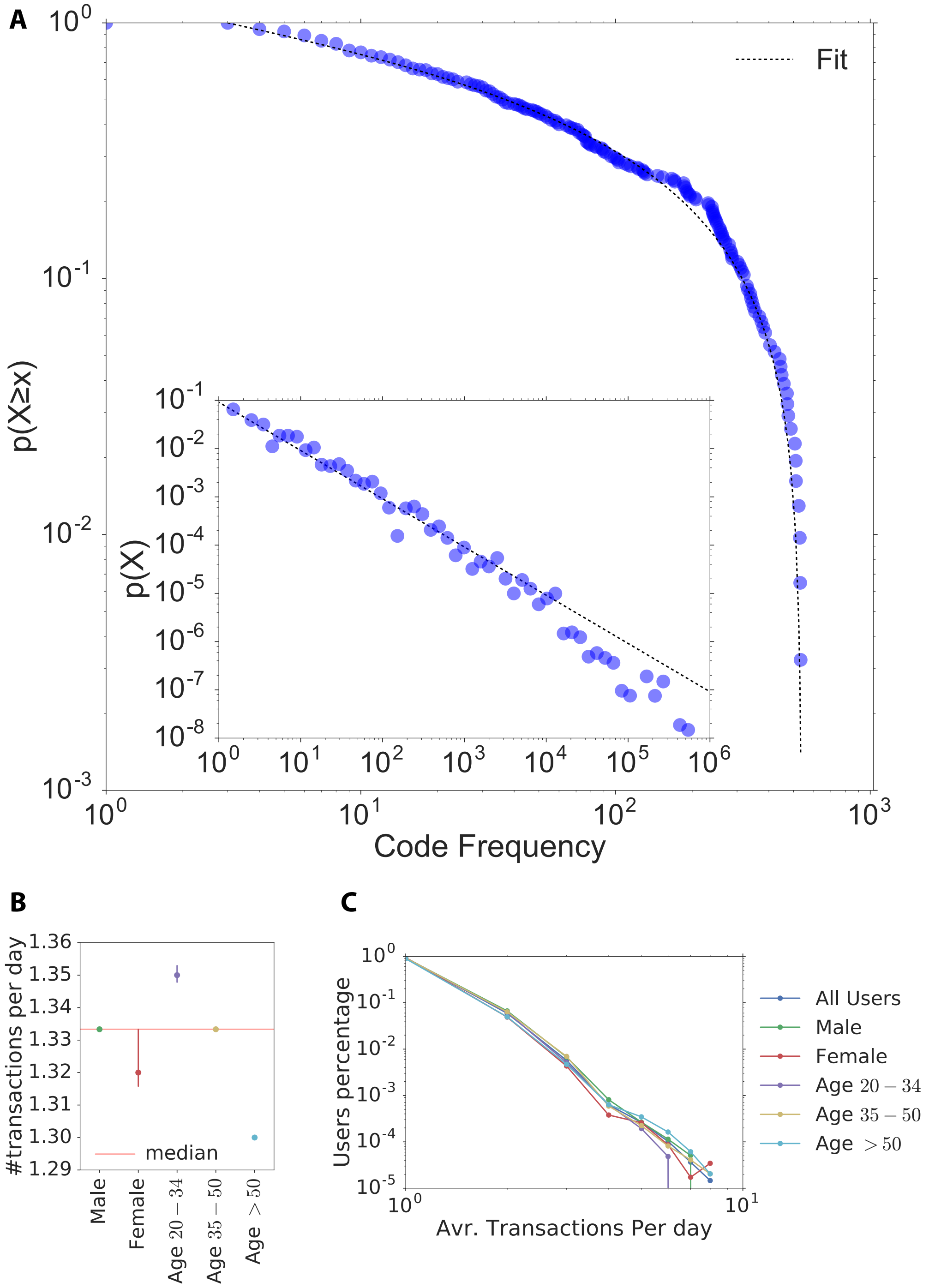

Supplementary Figure 2: **(A)** Complementary Cumulative Distribution and Probability Density Plot (inset) of MMCs' transaction codes. The probability distribution of a transaction code x presents Zipf's distribution $p(w_i) \propto x_i^{(-1.05)}$, with a Kolmogorov-Smirnov distance $D_{n=0.04}$ and with cutoff identified as the right-most point in the distribution before the fitted power law [1, 2]. **(B)** Median's distributions of the number of transactions per day divided by socio-demographic features; the error bars represent the confidence interval of 95% **(C)** Percentage of the number of transactions per day per user divided by socio- demographic features.

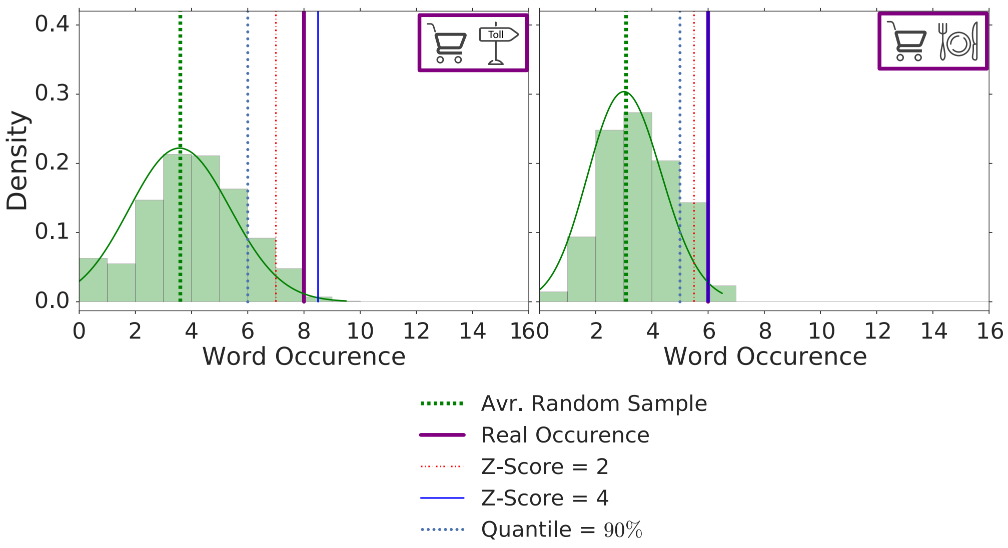

Supplementary Figure 3: Example of word occurrence distribution of the 1000 randomized code sequences preserving the same number of transactions per type. In both cases the real occurrence of the word showed in the purple box his higher than the average of the random sample. We can see that the z-score equal 2 computed from the sample in relation with the 90th quantile of the distribution. The icons used in this figure are work of Azaze11o/Shutterstock.com.

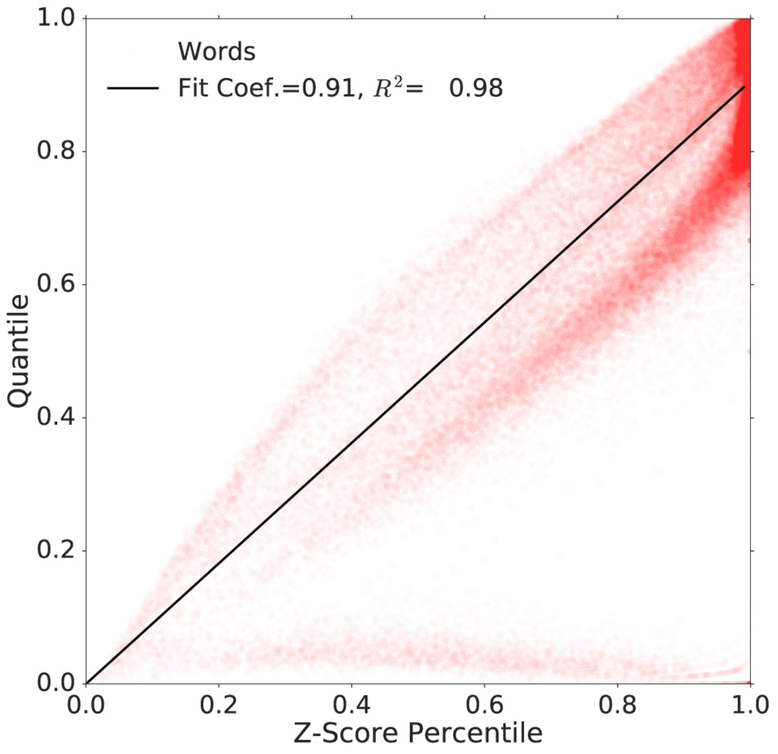

Supplementary Figure 4: Relation between the quantile value of the real occurrence of words respect to the randomized distribution vs. the z-score relative percentile of the words. The z-score relative percentile is highly correlated with the quantile position of the real word occurrence. We selected only the words with z-score>2, corresponding to the 97.73th percentile for a Gaussian distribution.

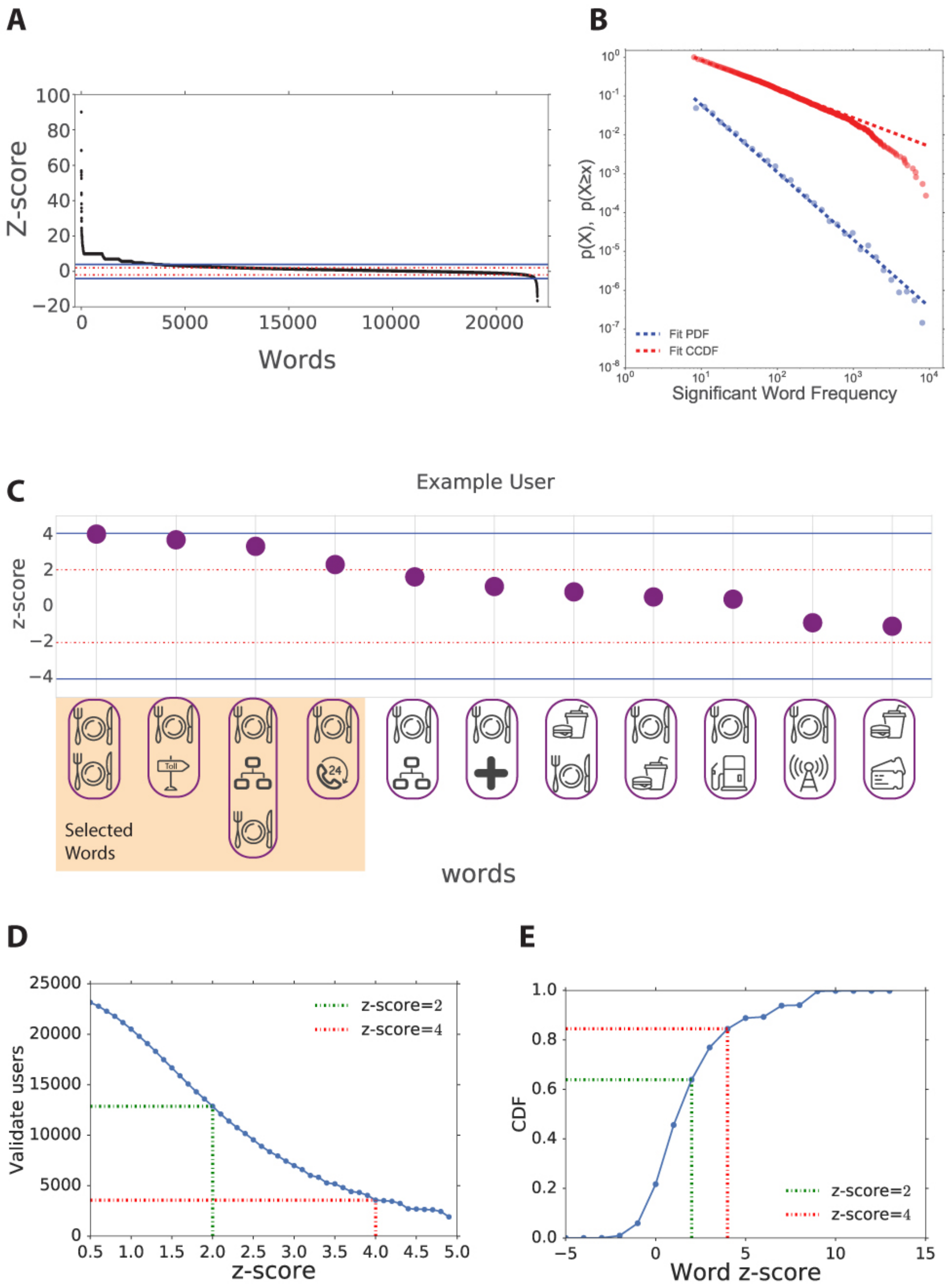

Supplementary Figure 5: **(A)** The z-score of each word depend on occurrence. The z-score of each word is calculated by comparing the code sequence with 1000 randomized code sequences for each user, preserving the number of transactions per type. **(B)** Probability Density Function plot of the occurrence of the significant words $\{W_i\}$ and its complementary cumulative distribution; the probability distribution words manifest a power law behavior $p(W_i) \propto x_i^{(-1.73)}$ with x_i frequency of the $\{W_i\}$ and Kolmogorov-Smirnov test of 0.02. **(C)** Z-score of a words occurrence for a sample user. We highlight in orange the users' associated set of words with a z-score greater than 2, that characterizes the user shopping routines. **(D)** Number of validate users given a z-score threshold. Selecting higher values of the z-score generate a decrease of analyzable users, while low values will impact the significance of the word selected. **(E)** CDF of the words z-scores. The icons used in this figure are work of Azaze11o/Shutterstock.com.

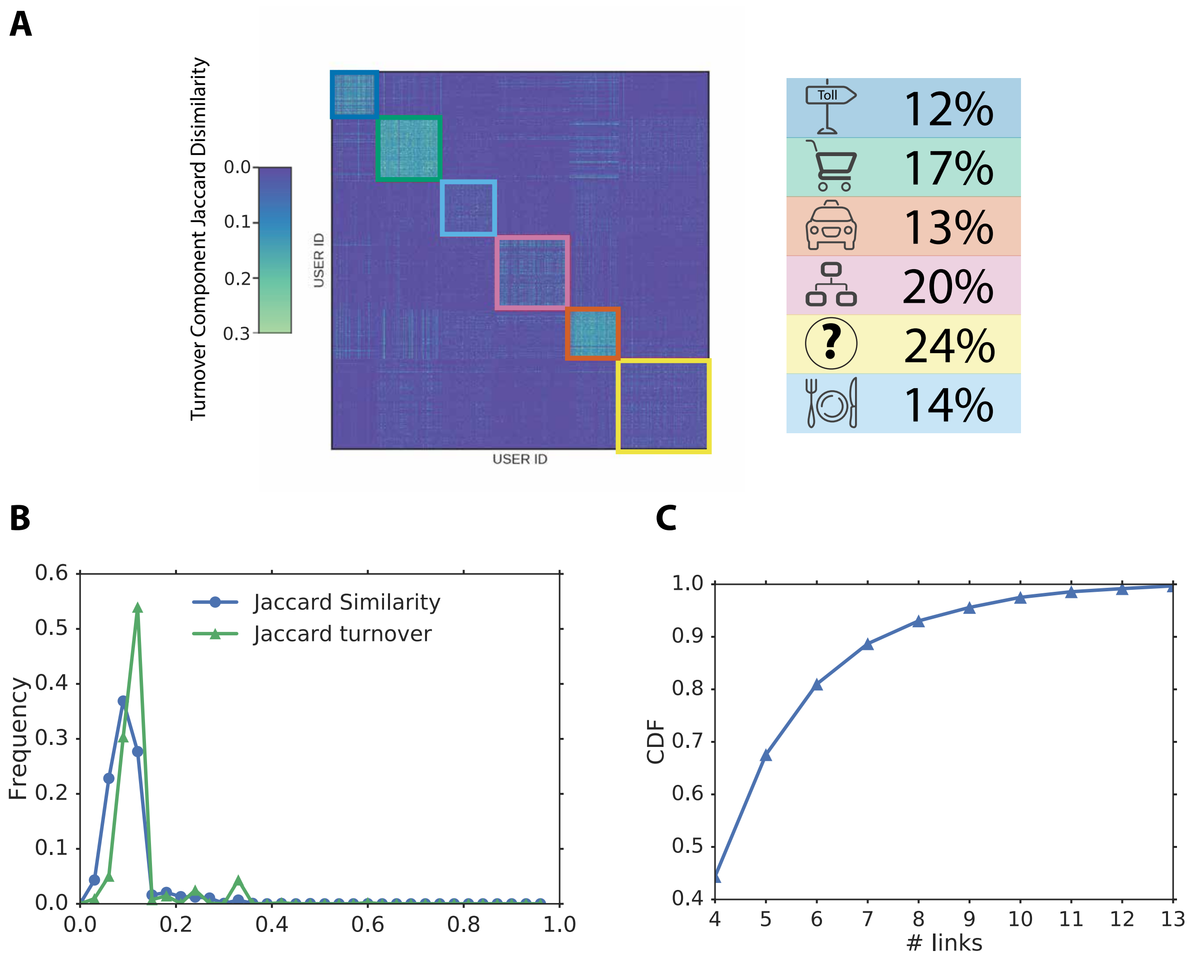

Supplementary Figure 6: **(A)** Clustering results using the turnover component of the Jaccard dissimilarity index [3]. **(B)** Distributions comparison between the Jaccard Similarity and the Jaccard turnover. **(C)** CDF of the number of links per users' network. The icons used in this figure are work of Azaze11o/Shutterstock.com.

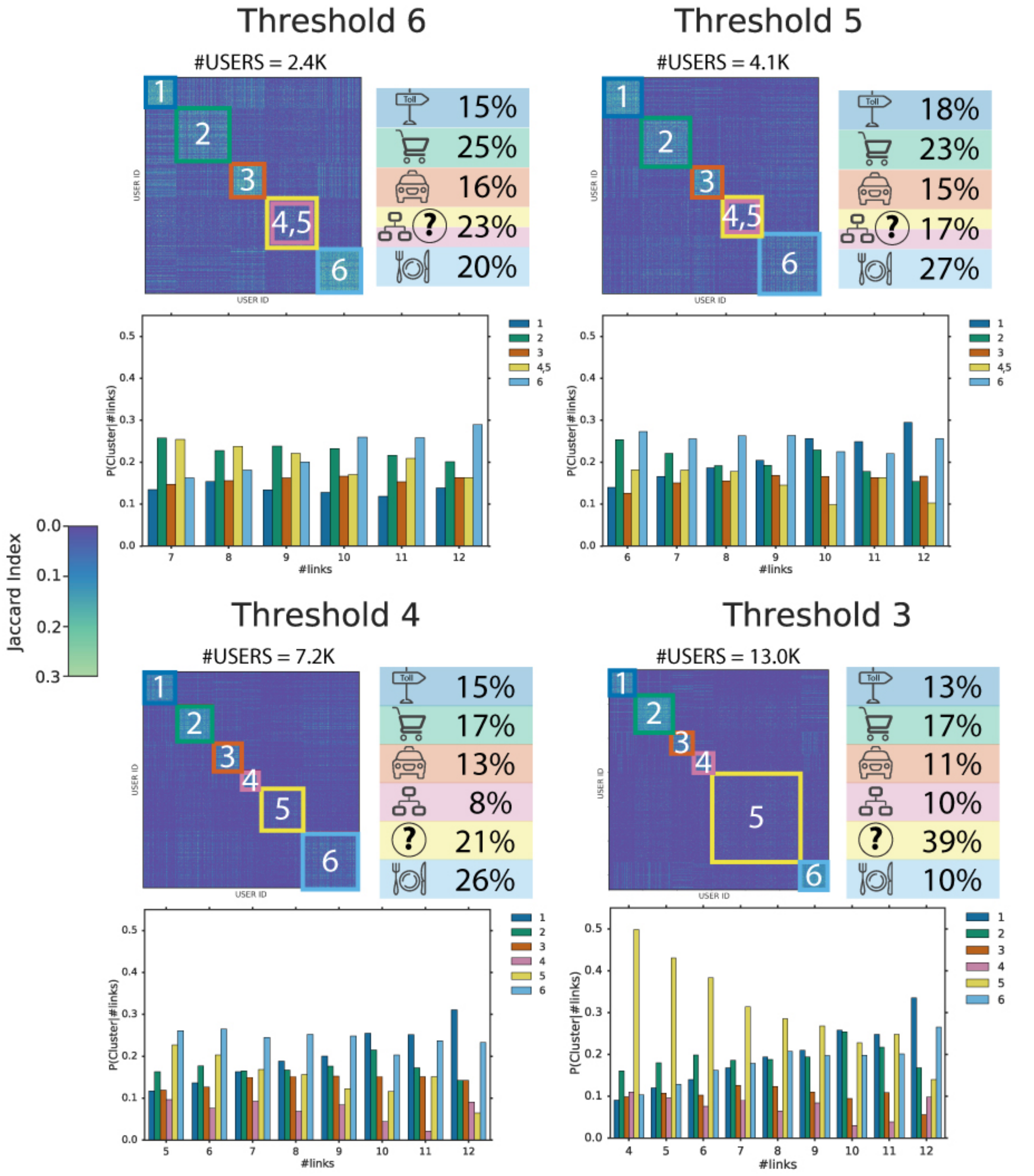

Supplementary Figure 7: Clustering results depending on the users' selection. For each threshold x we select all the users with more than x significant links. Using the Louvain Algorithm [4, 5] we perform the cluster of the users' similarity matrix of the selected users at each threshold. For each threshold, we show the proportion of users that belong to each cluster, the core transaction for that cluster (as defined in the main text, Fig. 3 main text) and the conditional probability for a user to belong in a given cluster depending on its number of significant links $P(cluster|\#links)$. By applying a lower threshold is it possible to increase the number of users analyzed. In particular, selecting users with more than 3 significant links improves the identification of clusters 4 and 6, which were misidentified when using higher thresholds. At the same time lowering the threshold increases the number of user that we are not able to categorize effectively (user percentage of cluster 5). The icons used in this figure are work of Azaze11o/Shutterstock.com.

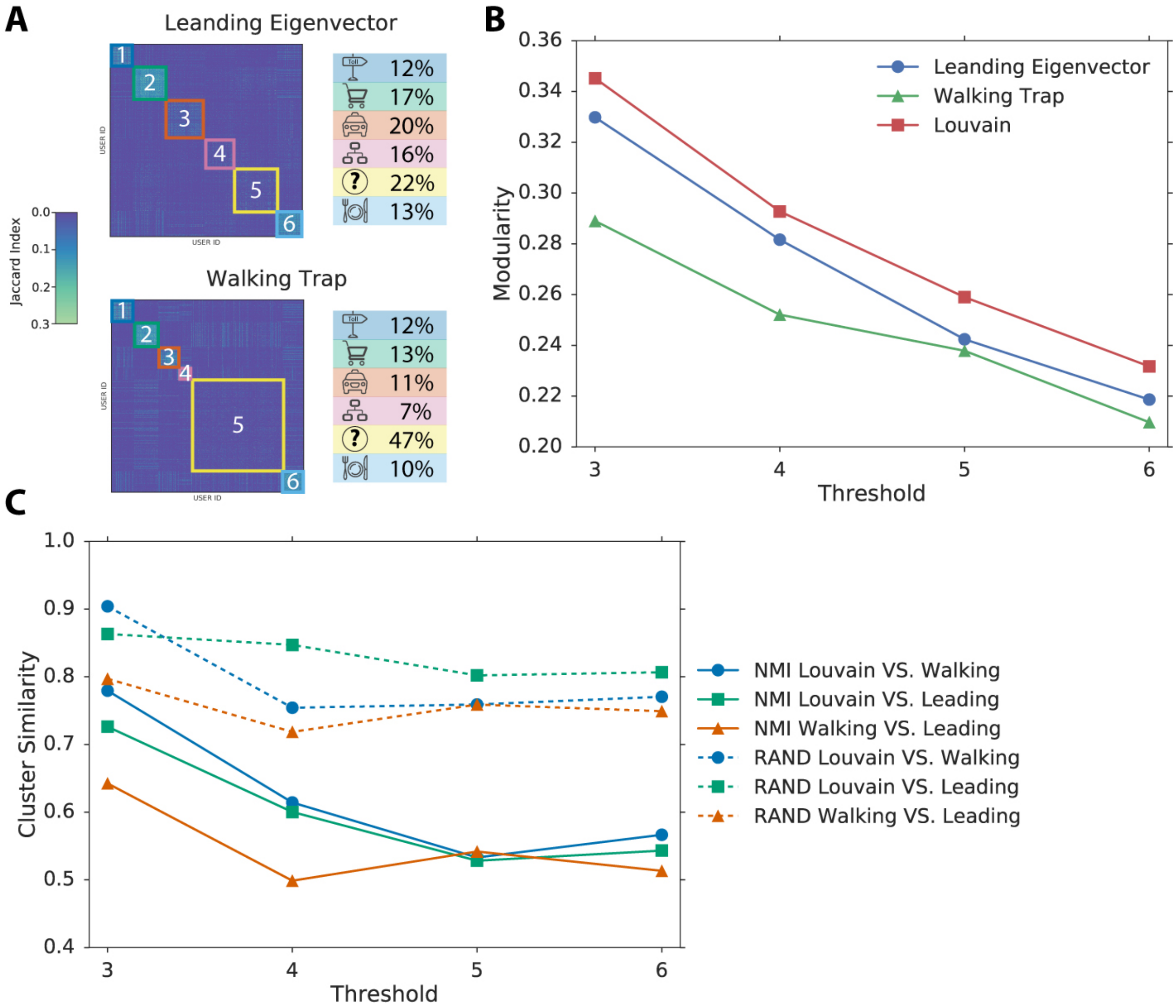

Supplementary Figure 8: **(A)** Cluster analysis of the users' similarity matrix for threshold 3 using the Leading Eigenvector [6] and Walking Trap [7] algorithms; both algorithms detect six different clusters as Louvain [4] (Fig. 3 main text and Supplementary Figure 5). **(B)** Network modularity analysis depending on the three cluster algorithms proposed. We see the Louvain algorithm always performs better in terms of modularity [8]. **(C)** Analysis of similarity between the three methods of data clustering performed, using Normalized Mutual Information (NMI) [9, 10] and Rand [11] index. Values near one suggest a higher similarity between the cluster identified by the Louvain algorithm and the other two algorithms. The icons used in this figure are work of Azaze11o/Shutterstock.com.

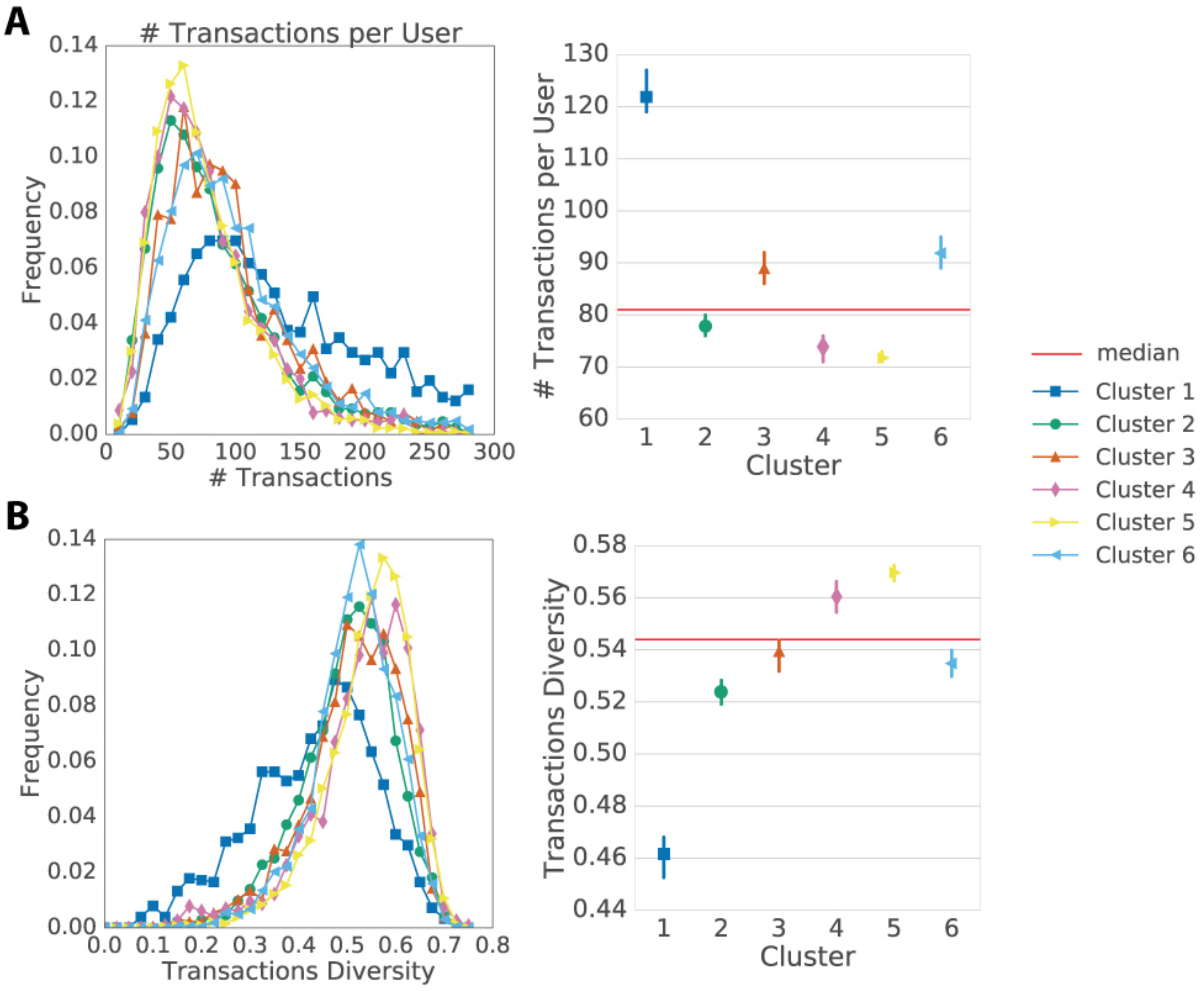

Supplementary Figure 9: **(A)** Distributions of the number of users' transactions per clusters (on the left), and confidence interval of 95% (on the right). **(B)** Distribution of the users' transaction diversity per clusters(on the left), and confidence interval of 95% (on the right). We measure the transaction diversity $D(i)$ of a user i by using the Shannon entropy of the user's transactions and dividing by the number of transactions N hence: $D(i) = [\sum_{t_i \in T_i} p(t_i) \log p(t_i)]/N$; with T the set of user transaction. The users identified as "commuters" (see main text) are those with low transactions diversity and a higher frequency of transactions. Conversely the users in the cluster 5 manifest a higher transaction diversity with a low number of transactions. These two factors combined means that the identification of the users' routines in this cluster is more challenging.

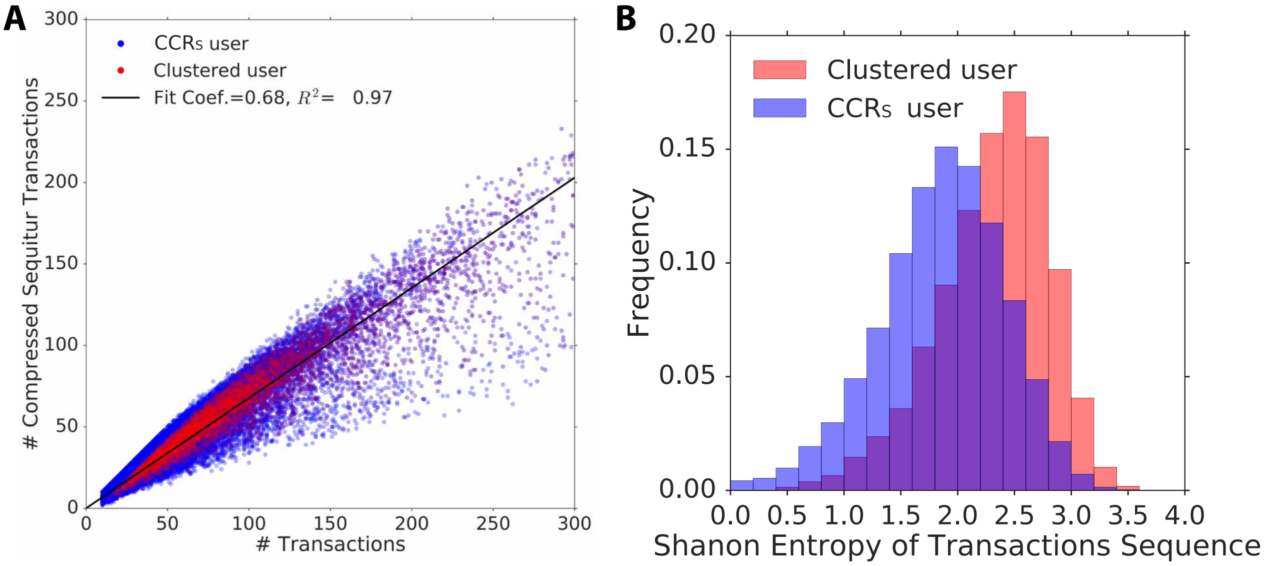

Supplementary Figure 10: **(A)** Sequitur compression ratio. Ratio between the original sequence transactions length and the length of Sequitur [12] sequence output. The compression ratio of the clustered user is 1.50. **(B)** Shanon entropy of the transactions Sequence. We define the Shannon entropy for a user i as $S(i) = [\sum_{t_i \in T_i} p(t_i) \log p(t_i)]$.

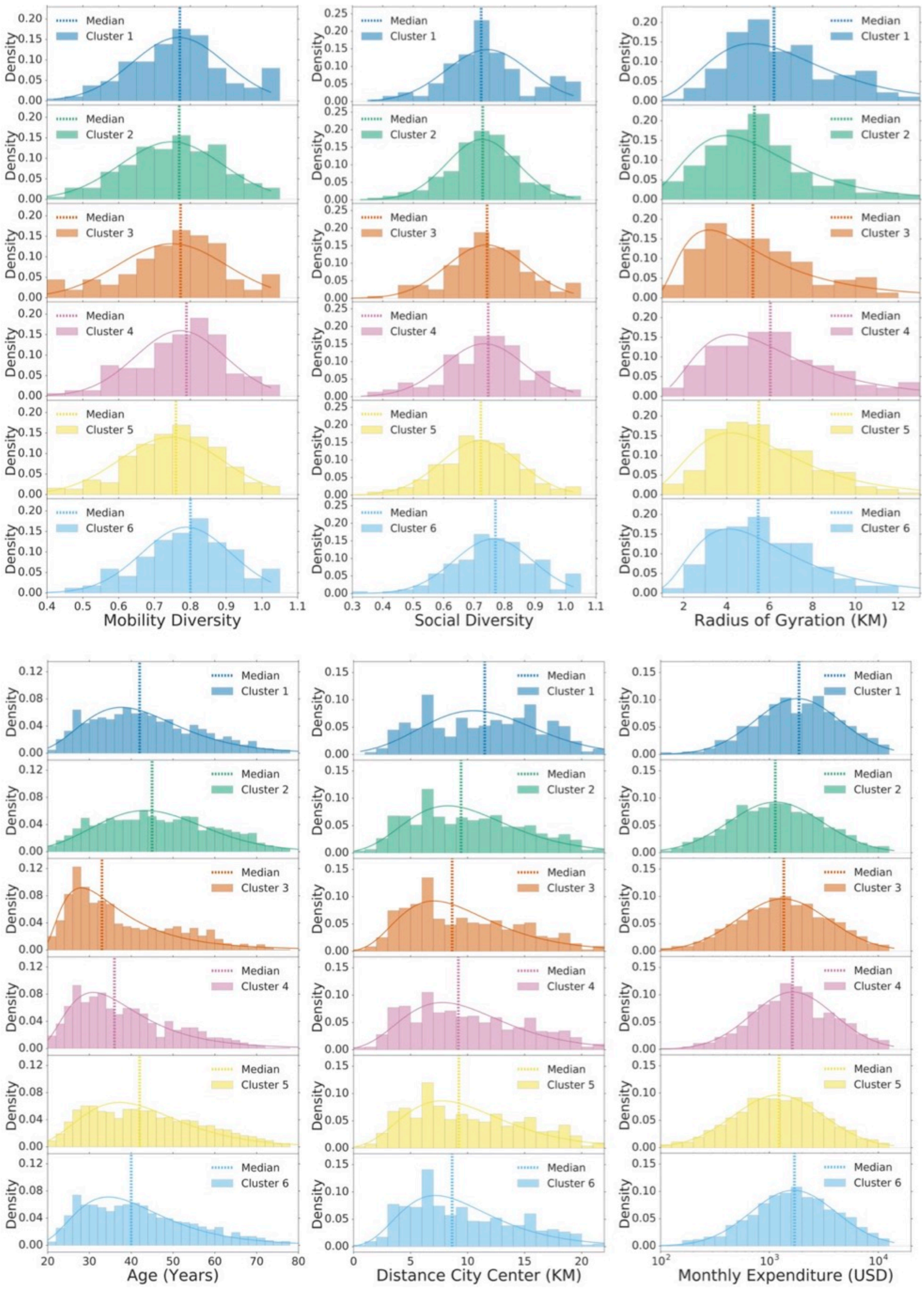

Supplementary Figure 11: Distributions of socio-demographic characteristics of individuals in each cluster.

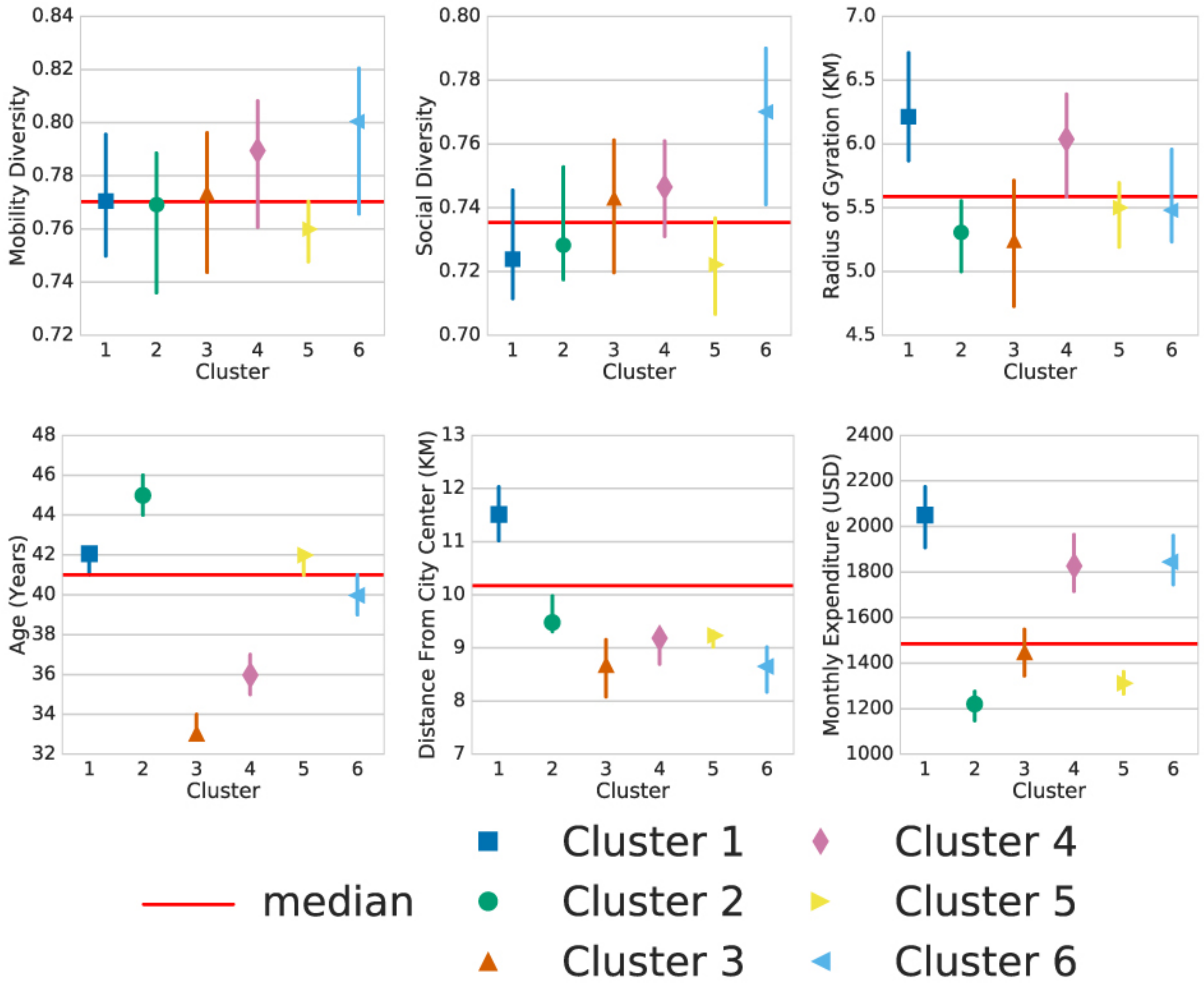

Supplementary Figure 12: Confidence intervals of 95% of socio-demographic characteristics of individuals in each cluster detected by our framework, and the solid in red representing the median values of the all clustered users.

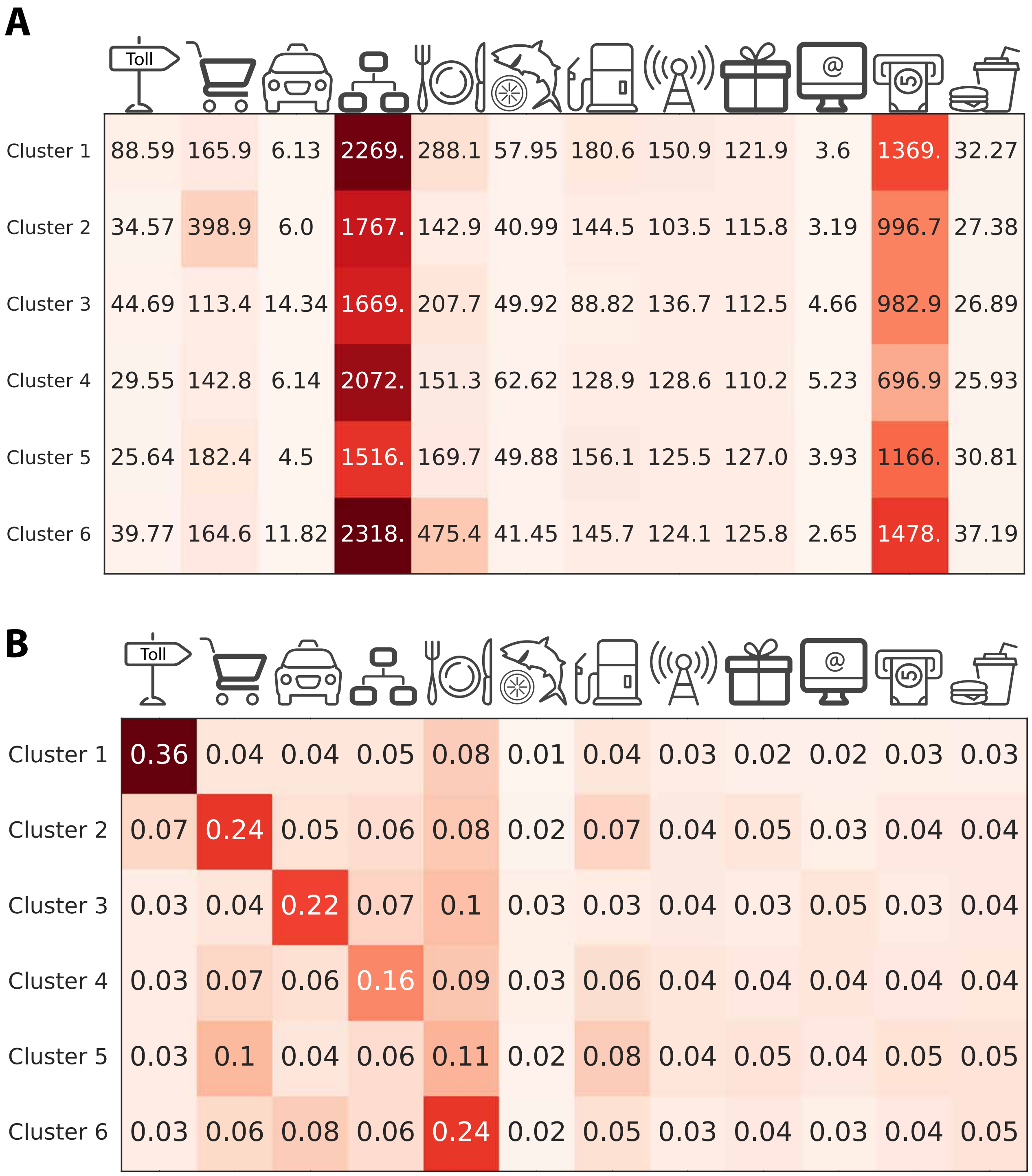

Supplementary Figure 13: **(A)** Median expenditure by transaction code (in USD for the 10 weeks considered). The overall clusters' expenditure are in agreement with the core transaction identified by our framework. **(B)** Frequency of transaction code for the 10 weeks considered for each fo the six cluster detected; the core transaction is a dominant feature for each of the clusters. Our method is able to extract information form a zipf like distribution uncovering behavior in shopping patterns. The icons used in this figure are work of Azaze11o/Shutterstock.com.

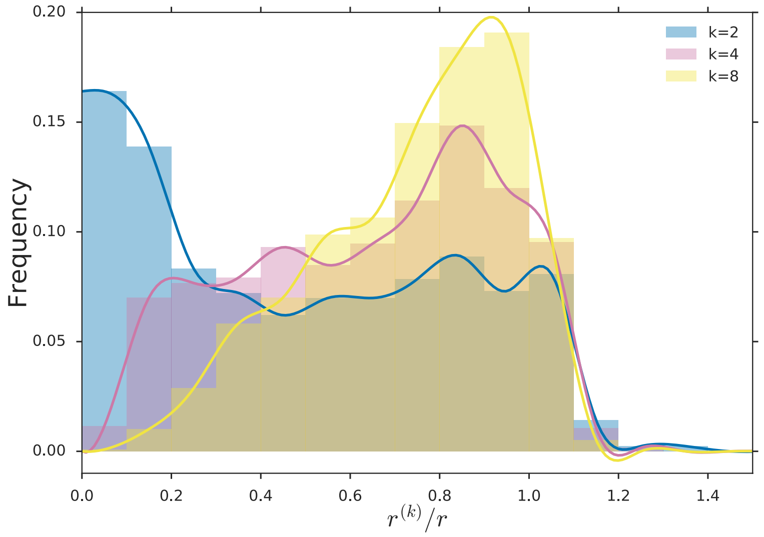

Supplementary Figure 14: Returners and Explorer [13] analysis all the users.

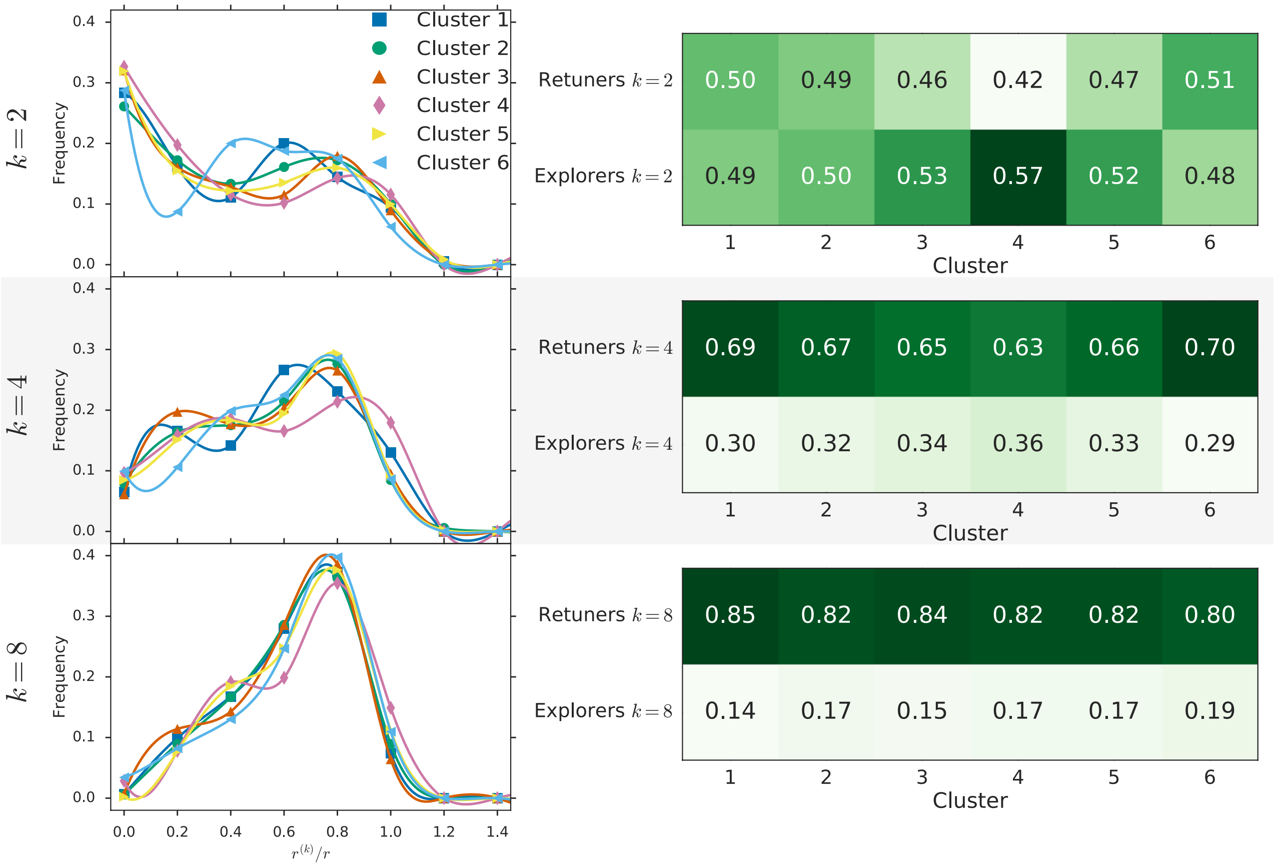

	1	2	3	4	5	6
Retuners $k=2$	0.50	0.49	0.46	0.42	0.47	0.51
Explorers $k=2$	0.49	0.50	0.53	0.57	0.52	0.48
Retuners $k=4$	0.69	0.67	0.65	0.63	0.66	0.70
Explorers $k=4$	0.30	0.32	0.34	0.36	0.33	0.29
Retuners $k=8$	0.85	0.82	0.84	0.82	0.82	0.80
Explorers $k=8$	0.14	0.17	0.15	0.17	0.17	0.19

Supplementary Figure 15: Returners and Explorer [13] analysis by clusters.

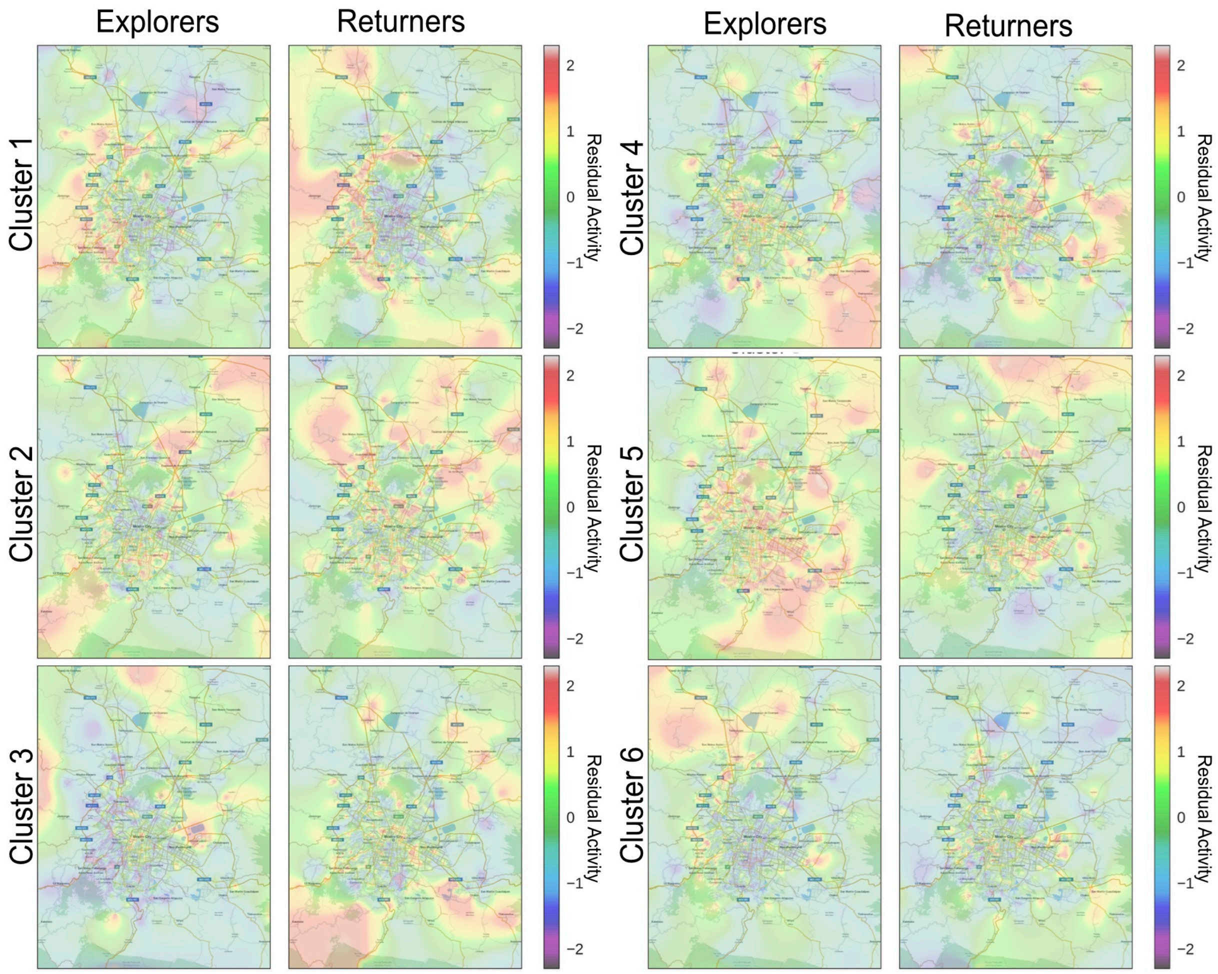

Supplementary Figure 16: Returners and Explorer [13] cell tower residual activity [14]. The maps were created using the software Mathematica

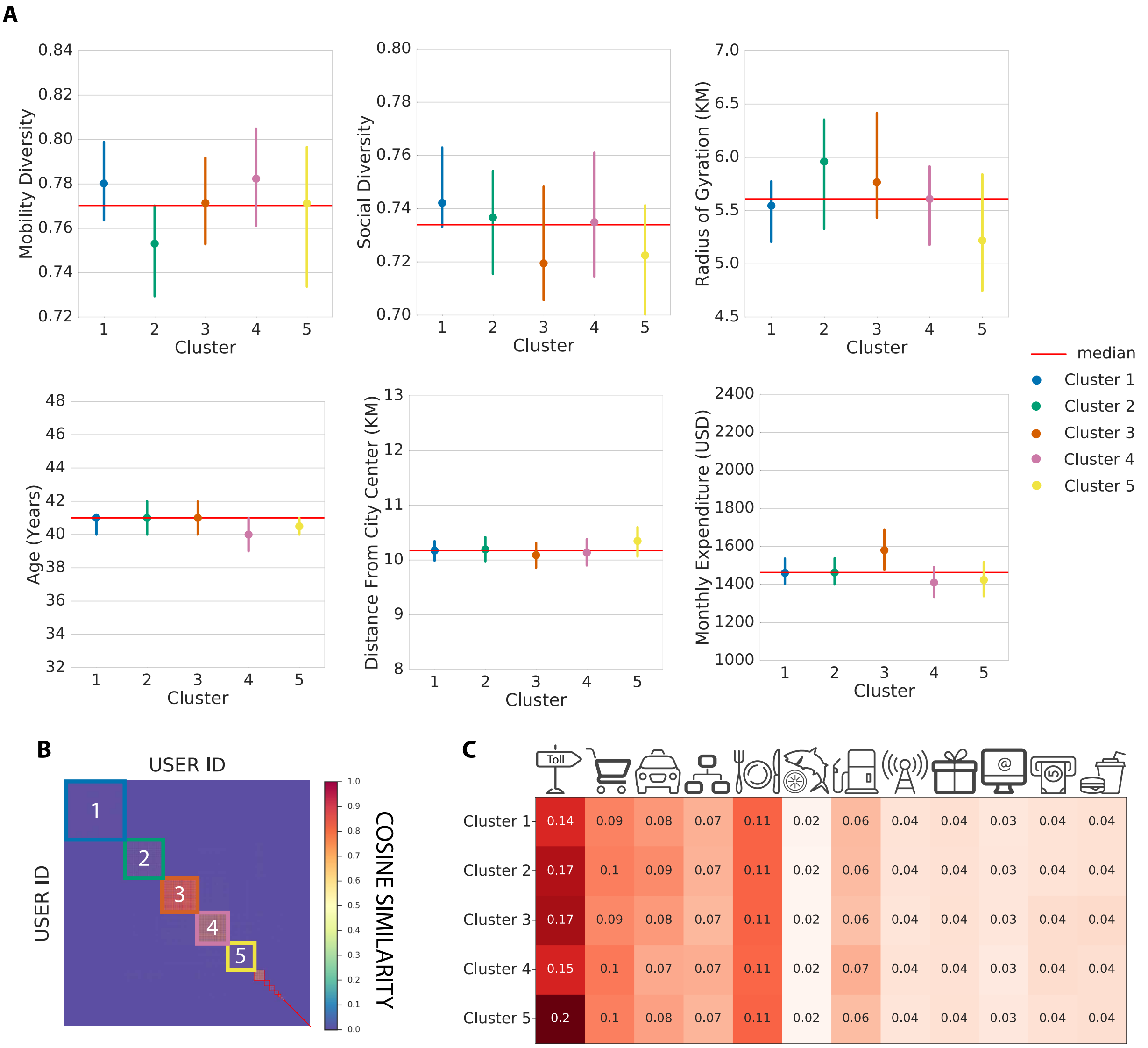

Supplementary Figure 17: Cluster analysis of the selected user using the TF-IDF [15, 16] algorithm to compare the users transaction code sequence. **(A)** Confidence interval of 95% of socio-demographic characteristics of the five clusters detected. The TF-IDF is not able to capture any hidden information form the zipf like distribution of the credit card transaction. According with our socio-demographic metrics each of the 5 cluster detected is only a random sample of users and do not show any particular behavior. **(B)** Cluster analysis of the 13.0K selected users. The Louvain algorithm has been performed over the users' cosine similarity matrix of the TF-IDF, with a threshold at 0.6 of cosine similarity [15]. The clusters 3,4 show and high users' similarity in the TF-IDF without showing any meaningful socio-demographic relation. **(C)** Transactions frequency for each clusters show the same zipf like distributions outlining that standard methods are not suitable to extract information form a zipf like distribution as our framework (see Supplementary Figure 13B). The icons used in this figure are work of Azaze11o/Shutterstock.com.

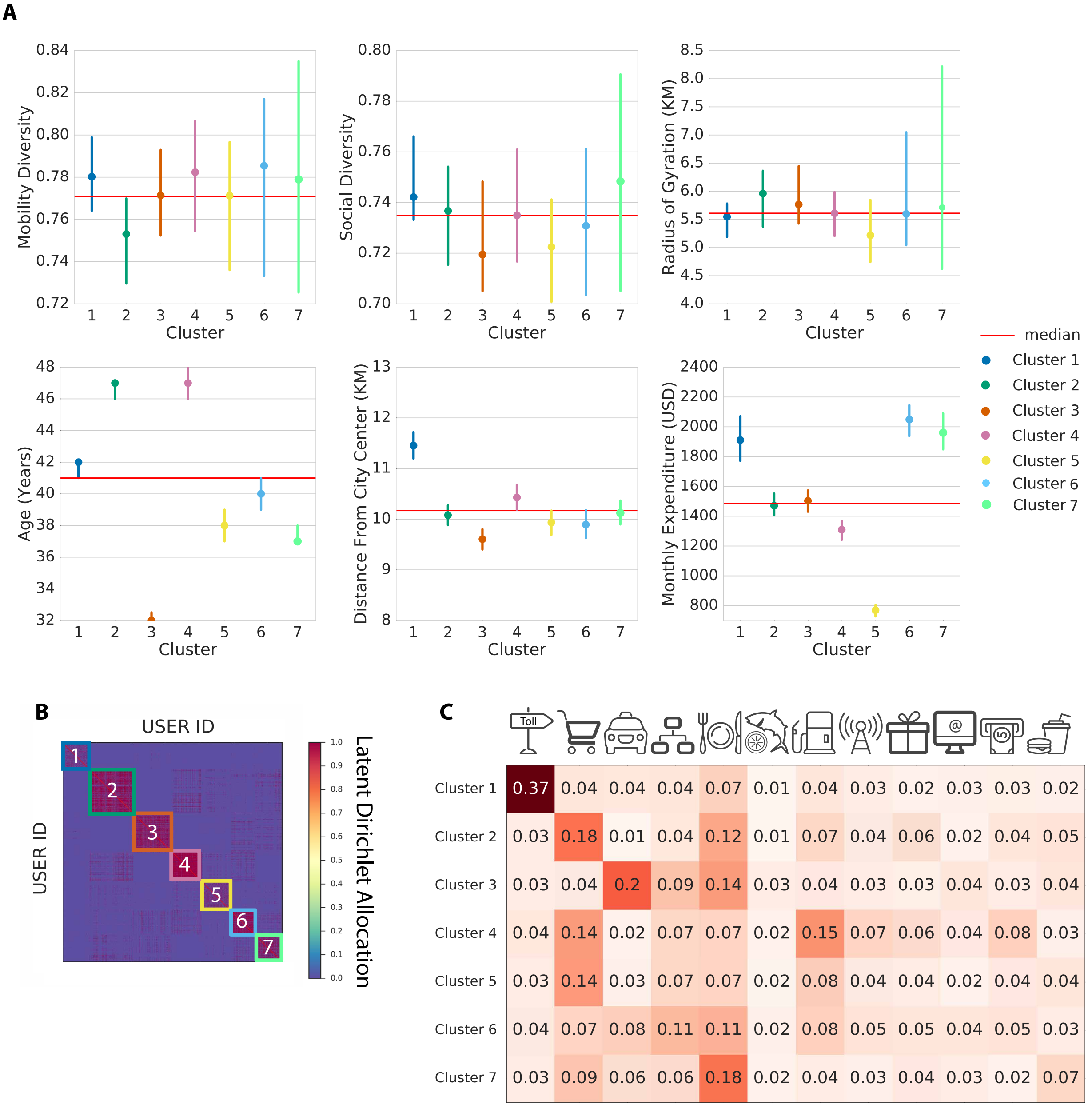

Supplementary Figure 18: Cluster analysis of the selected user using Latent Dirichlet Allocation (LDA) [17] to model user transactions. **(A)** Confidence interval of 95% of socio-demographic characteristics of the seven clusters detected the solid in red representing the median values of the all clustered users. **(B)** Cluster analysis of the 13.0K selected users. Using LDA we model each user as a mixture of five spending behaviors, where each behavior is a mixture of transaction codes. We compute the Jensen-Shannon divergence [18] for the user similarity matrix, then perform the Louvain algorithm with a threshold of 0.1. We compare the clusters detected with the LDA and the Sequitur methods using the Normalized Mutual Information [9, 10] NMI = 0.2 and the Rand=0.7 [11]. This two tests show a degree of similarity among the clusters. **(C)** Frequency of the transaction codes for the 10 weeks considered for each of the seven clusters detected. The clusters extracted with the LDA manifest similar characteristics with the ones extracted with our method (Supplementary Figure 13B). The (1,2,3,7) clusters detected by LDA share distribution in spending codes with the (1,2,3,6) cluster of the sequitur. Moreover, the clusters (4,5) of LDA are very similar at the sequitur cluster 5. The icons used in this figure are work of Azaze11o/Shutterstock.com.

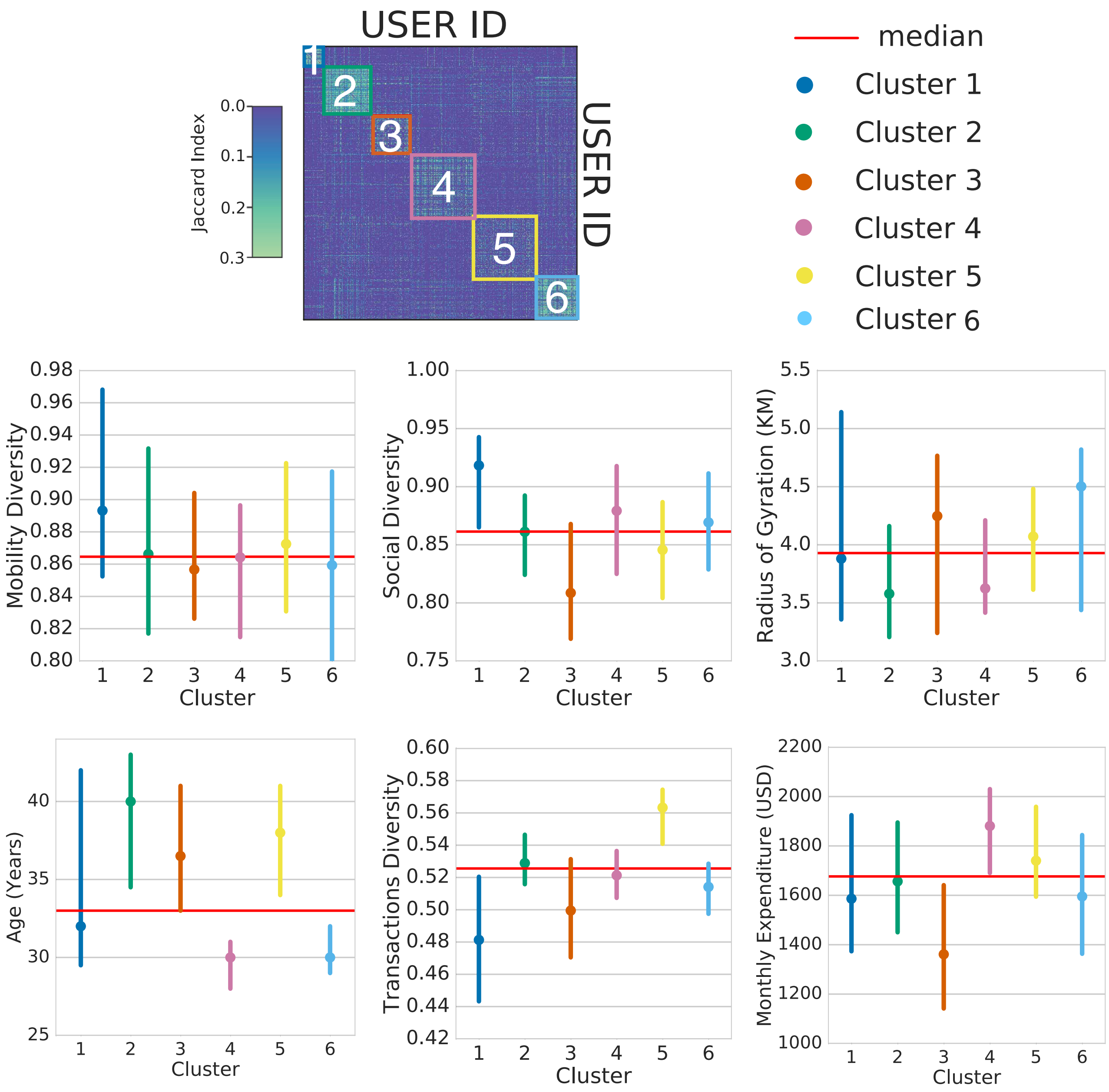

Supplementary Figure 19: **Cluster analysis for the second city analyzed in Mexico: Puebla.** Confidence interval of 95% of socio-demographic characteristics. In this city the users have an higher mobility and social diversity with low radius of gyration. (For further considerations see Supplementary Figure 20-21)

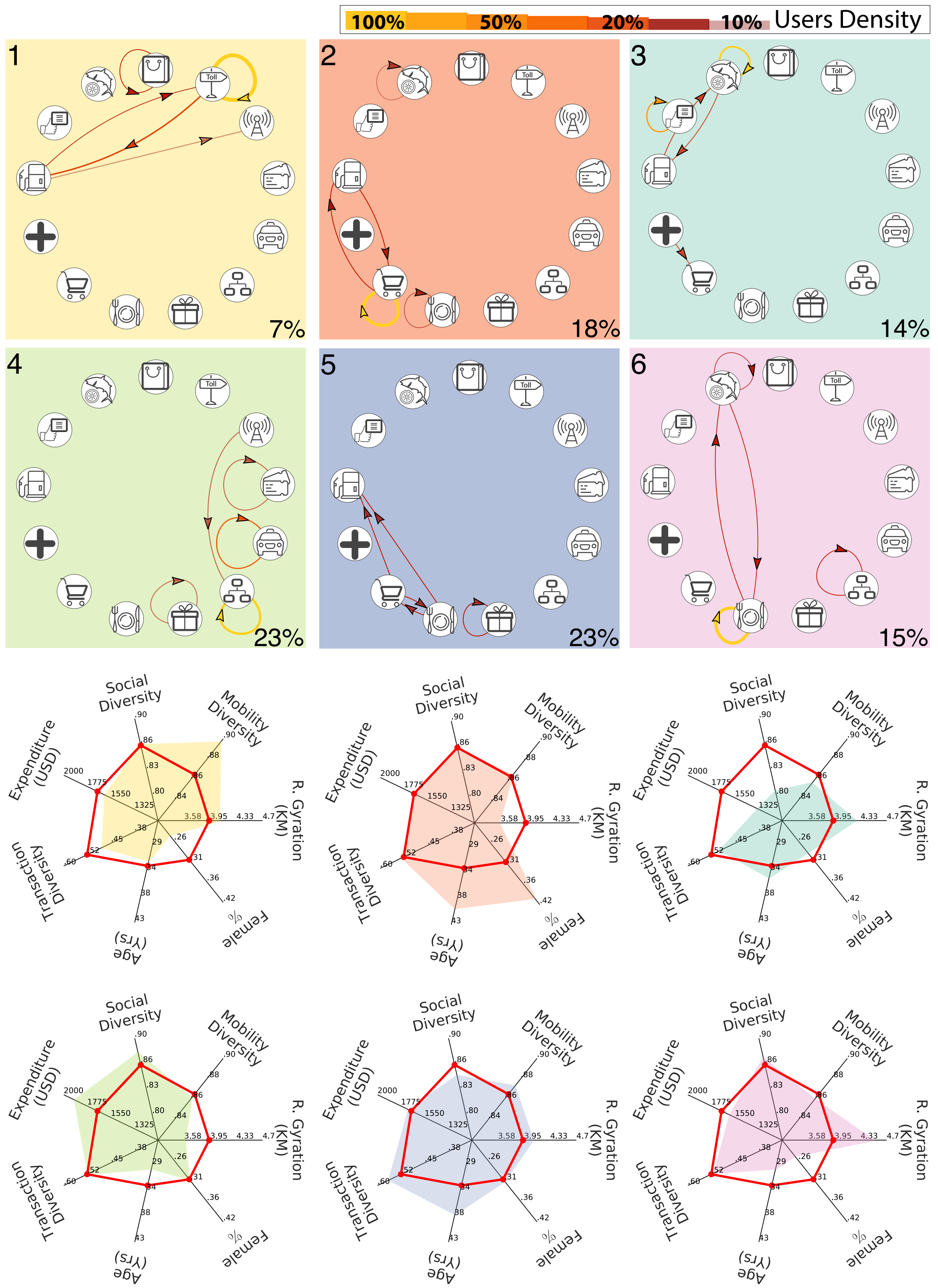

Supplementary Figure 20: **Groups based on their spending habits for the second city analyzed in Mexico: Puebla.** We show the top 5 most frequent spending sequences of the users in each group, representing more than 30% of users' shopping routines. The percentage of the total users in each group is shown in the bottom-right corner. Distribution of individual characteristics among users: gender radius of gyration, mobility diversity, social diversity, median expenditure by month, transaction diversity and age. While the clusters (1,2,4,5,6), manifest similarity among the two cities. The cluster 3 in the City B has different routines with the core transactions in Miscellaneous Food store and insurance instead of taxi and restaurants (see Supplementary Figure 21 for further comparison on the socio-demographic-mobility indicators between the two cities). The icons used in this figure are work of Azaze11o/Shutterstock.com.

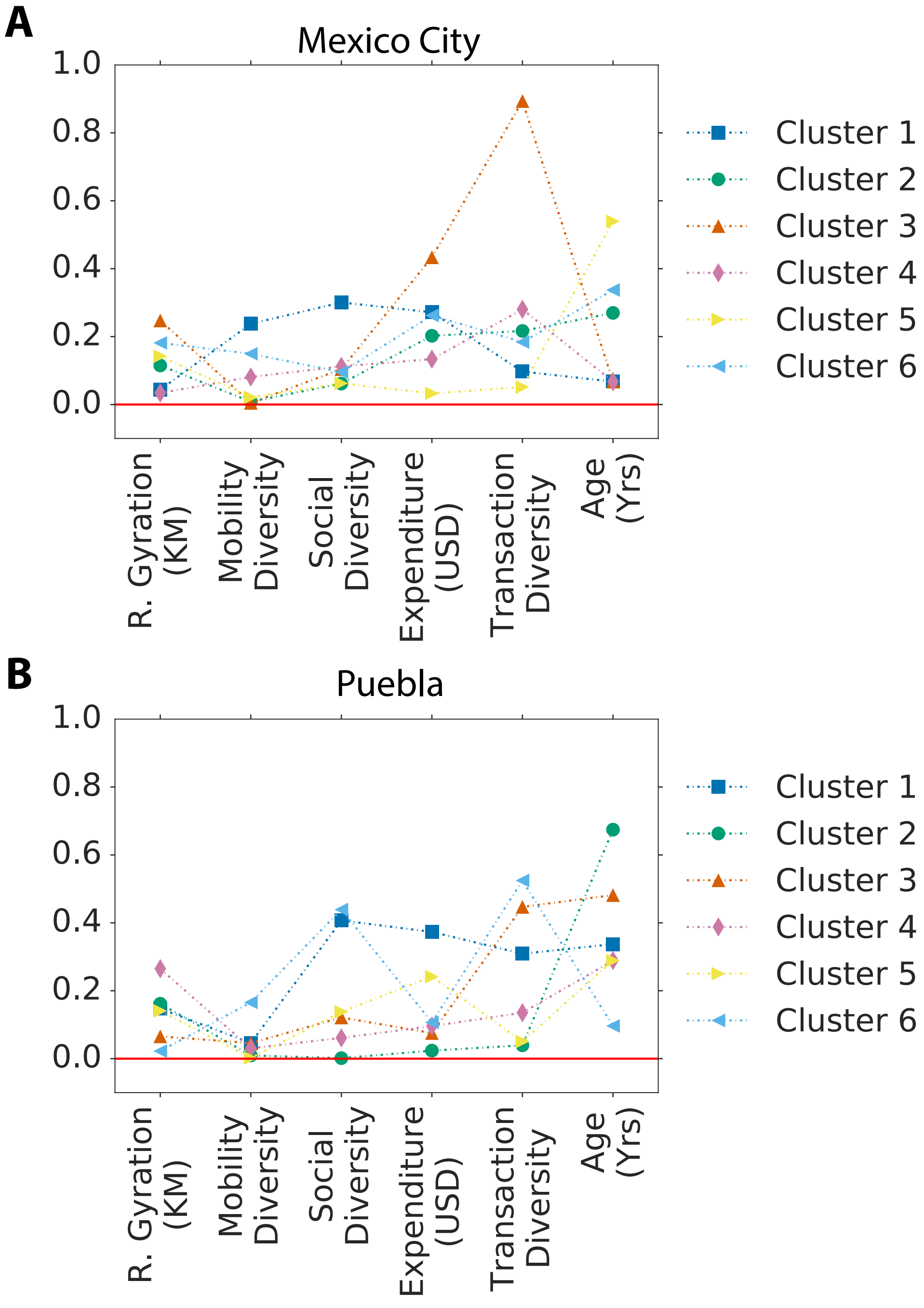

Supplementary Figure 21: **Analysis of median socio-demographic-mobility index variation per cluster**. The y axis represents $(\tilde{x}_i - \tilde{X})/\text{MAD}(X)$; with $\tilde{X}$ the median of the whole dataset for the socio-demographic-mobility attribute X, $\tilde{x}_i$ the median of the same socio-demographic-mobility attribute of the i-cluster users and MAD the Median Absolute Deviation. Remarkably the behaviors of the clusters (2,4,5,6) are very similar between the two cities considered. The two Clusters 3 as already stress represent two different segments of the population. Meanwhile the clusters 1 of the commuters have different behaviors maintaining the lower transaction diversity this could be due to the different topology of the cities.

Supplementary References

[1] Alstott, J., Bullmore, E. & Plenz, D. powerlaw: a python package for analysis of heavy-tailed distributions. *PloS one* **9**, e85777 (2014).

[2] Clauset, A., Shalizi, C. R. & Newman, M. E. Power-law distributions in empirical data. *SIAM review* **51**, 661–703 (2009).

[3] Baselga, A. The relationship between species replacement, dissimilarity derived from nestedness, and nestedness. *Global Ecology and Biogeography* **21**, 1223–1232 (2012).

[4] Blondel, V. D., Guillaume, J.-L., Lambiotte, R. & Lefebvre, E. Fast unfolding of communities in large networks. *Journal of statistical mechanics: theory and experiment* **2008**, P10008 (2008).

[5] Staudt, C. L. & Meyerhenke, H. Engineering parallel algorithms for community detection in massive networks. *IEEE Transactions on Parallel and Distributed Systems* **27**, 171–184 (2016).

[6] Newman, M. E. Finding community structure in networks using the eigenvectors of matrices. *Physical review E* **74**, 036104 (2006).

[7] Pons, P. & Latapy, M. Computing communities in large networks using random walks. In *International symposium on computer and information sciences*, 284–293 (Springer, 2005).

[8] Newman, M. E. Modularity and community structure in networks. *Proceedings of the national academy of sciences* **103**, 8577–8582 (2006).

[9] Danon, L., Diaz-Guilera, A., Duch, J. & Arenas, A. Comparing community structure identification. *Journal of Statistical Mechanics: Theory and Experiment* **2005**, P09008 (2005).

[10] Ana, L. & Jain, A. K. Robust data clustering. In *Computer Vision and Pattern Recognition, 2003. Proceedings. 2003 IEEE Computer Society Conference on*, vol. 2, II–II (IEEE, 2003).

[11] Rand, W. M. Objective criteria for the evaluation of clustering methods. *Journal of the American Statistical association* **66**, 846–850 (1971).

[12] Nevill-Manning, C. G. & Witten, I. H. Identifying hierarchical structure in sequences: A linear-time algorithm. *Journal of Artificial Intelligence Research* **7**, 67–82 (1997).

[13] Pappalardo, L. *et al.* Returners and explorers dichotomy in human mobility. *Nature communications* **6**, 8166 (2015).

[14] Toole, J. L., Ulm, M., González, M. C. & Bauer, D. Inferring land use from mobile phone activity. In *Proceedings of the ACM SIGKDD international workshop on urban computing*, 1–8 (ACM, 2012).

[15] Roque, F. S. *et al.* Using electronic patient records to discover disease correlations and stratify patient cohorts. *PLoS computational biology* **7**, e1002141 (2011).

[16] Hidalgo, C. A., Blumm, N., Barabási, A.-L. & Christakis, N. A. A dynamic network approach for the study of human phenotypes. *PLoS computational biology* **5**, e1000353 (2009).

[17] Blei, D. M., Ng, A. Y. & Jordan, M. I. Latent dirichlet allocation. *Journal of machine Learning research* **3**, 993–1022 (2003).

[18] Lin, J. Divergence measures based on the shannon entropy. *IEEE Transactions on Information theory* **37**, 145–151 (1991).
